# Phosphorylation of STAT3 Promotes Vasculogenic Mimicry by Inducing Epithelial-to-Mesenchymal Transition in Colorectal Cancer

**DOI:** 10.1177/1533034617742312

**Published:** 2017-11-22

**Authors:** Cong Han, Baocun Sun, Xiulan Zhao, Yanhui Zhang, Qiang Gu, Fang Liu, Nan Zhao, Lili Wu

**Affiliations:** 1 Department of Pathology, Tianjin Medical University, Tianjin, People’s Republic of China; 2 Department of Pathology, General Hospital of Tianjin Medical University, Tianjin, People’s Republic of China; 3 Department of Pathology, Cancer Hospital of Tianjin Medical University, Tianjin, People’s Republic of China

**Keywords:** colorectal cancer, p-STAT3, vasculogenic mimicry, IL6, AG490

## Abstract

Vasculogenic mimicry refers to the process by which highly invasive cancer cells mimic endothelial cells by forming blood channels. Vasculogenic mimicry is important for the invasion and metastasis of tumor cells in colorectal cancer. STAT3 was initially identified as a mediator of the inflammation-associated acute phase response. The phosphorylation of Signal Transducers and Activators of Transcription 3 (p-STAT3) is closely related to tumor invasion and migration. We analyzed the relationship between p-STAT3 and vasculogenic mimicry formation in 65 human colorectal cancer samples, and the results showed that the expression of p-STAT3 is significantly correlated with vasculogenic mimicry, tumor metastasis, Tumor, Lymph Node and Metastasis Stage (TNM Stage), and poor prognosis. It is known that interleukin 6 can induce the phosphorylation of STAT3. We found that using interleukin 6 to induce p-STAT3 activation in colorectal cancer cell lines can result in vasculogenic mimicry and using AG490 to suppress p-STAT3 activation restrained vasculogenic mimicry. Furthermore, the state of p-STAT3 activation can affect epithelial-to-mesenchymal transition. By immunofluorescence double staining, we discovered that p-STAT3 expression is more directly correlated with the epithelial-to-mesenchymal transition marker vimentin than with the vasculogenic mimicry-related protein VE-cadherin. These data show that activated p-STAT3 upregulates epithelial-to-mesenchymal transition–related proteins and promotes vasculogenic mimicry.

## Introduction

Colorectal cancer (CRC) is the third most frequently diagnosed cancer worldwide, and most patients are diagnosed with advanced disease.^1^ Indeed, the accepted principle underlying tumor survival and metastatic potentials has been the tumor microenvironment and vascular network formation.^2^ It is known that endothelial cells can form blood vessels. However, angiogenesis is not the only process by which tumors acquire their blood supply. Malignant tumor cells can line up to form functional blood vessels in a mechanism called vasculogenic mimicry (VM).^2,3^

Vasculogenic mimicry refers to the process by which highly invasive cancer cells mimic endothelial cells by forming blood channels, which was first reported as a nonangiogenesis-dependent pathway in malignant melanoma in 1999.^4^ Since then, VM has been reported in many aggressive tumors, such as head and neck tumors, breast cancer, ovarian carcinoma, hepatocellular carcinoma, gastric carcinoma, and lung cancer.^5–9^ We and other researchers have also observed this phenomenon in CRC.^1^ These findings explain why a series of drugs once heralded as game changers in cancer treatment were less effective than hoped.

Signal Transducers and Activators of Transcription 3 (STAT3), a key member of the Janus-Actived Kinase / Signal Transducers and Activators of Transcription (JAK/STAT) pathway, is constitutively activated in CRC,^10^ and evidence has supported its role in mediating cancer cell invasion and migration.^11,12^ Interleukin 6 (IL-6) can induce the phosphorylation of STAT3 to p-STAT3^11,13^ and p-STAT3 is the primary activated form of STAT3.^14^ Jak is responsible for the tyrosine phosphorylation of STAT3 in response to extracellular signals and oncogenes.^15^ The newly described Jak inhibitor AG490 blocks the constitutive activation of STAT3.^16^ Therefore, AG490 has been used to inhibit the activation of STAT3 in cancer cell lines.^17^

In this study, we focused on exploring the potential links between p-STAT3 activation and VM formation. Here, we demonstrate that the overexpression of p-STAT3 is correlated with poor prognosis in patients and the presence of VM in human CRC. We found that p-STAT3 activation regulated VM formation and induced epithelial-to-mesenchymal transition (EMT) *in vitro*. Furthermore, our data indicate that p-STAT3 activation exercises a great influence on VM by affecting the EMT marker vimentin more than by directly acting on the VM marker VE-cadherin. Therefore, inhibiting the phosphorylation of STAT3 might serve as a therapeutic method for inhibiting VM formation and improving the prognosis of CRC.

## Materials and Methods

### Tissue Samples

In the current study, 65 formalin-fixed, paraffin-embedded human CRC tissue samples were collected at the General Hospital of Tianjin Medical University from January 2002 to December 2005. None of the patients received chemotherapy or radiotherapy before operation. Clinical parameter data were obtained from the patients’ clinical records. The pathological diagnosis of all cases was independently verified by 2 senior pathologists by observing the H&E sections.

### Chemicals and Reagents

The details are provided in the Supplemental Material.

### Immunohistochemical Staining and CD31/Periodic Acid–Schiff Double Staining

For the immunohistochemical staining, the tissue sections were deparaffinized, hydrated, and rehydrated according to standard protocols. Antigen retrieval was performed, and nonspecific binding sites were blocked. The sections were then incubated with the primary antibody overnight at 4°C and with the secondary antibody for 30 minutes at 37°C. The color was developed using 3,3′-diaminobenzidine chromogen and counterstained with hematoxylin.

For the CD31/periodic acid–Schiff (PAS) double staining, immunohistochemical staining methods were performed as above, and for CD31 staining, the sections were subjected to PAS staining and counterstained with hematoxylin.

### Cell Culture and Proliferation Assay

Human CRC cell lines, HCT116 and HT29, were purchased from the Chinese Academy of Medical Sciences Basic Medical Cell Resource Center. The HCT116 cells were cultured in ISCOVE's Modified Dulbecco’s Modified Eagle Medium (IMDM) supplemented with 10% Fetal Bovine Serum (FBS) under standard culture conditions (37°C, 95% humidified air, and 5% CO_2_). The HT29 cells were cultured in Dulbecco’s Modified Eagle Medium: Nutrient Mixture F-12 (DMEM/F12) medium supplemented with 5% FBS under standard culture conditions.

To activate STAT3 phosphorylation, the CRC cells were treated with 0, 10, 20, 30, 40, or 50 ng/mL IL-6 for 48 hours. To inhibit STAT3 phosphorylation, the cells were treated with 0, 10, 20, 30, 40, or 50 μM AG490 for 48 hours. For IL-6 treatment, the CRC cells were incubated with 20 ng/mL and for AG490 treatment with 10 μM for 48 hours before harvesting for measurements.

### 3- (4,5-dimethyl-2-thiazolyl)-2,5-diphenyl-2-H-tetrazolium bromide Assay (MTT Assay)

Human CRC cells (9000 cells/well) with different dilution of IL-6 or AG490 treatment were placed in 96-well plates and continually cultured for different periods of time (24,48, and 72 hours). Ten microliter of 0.5 mg/mL MTT solution was added to each well, and the plate was further incubated at 37°C for 4 hours. Then the medium was removed, and the precipitated formazan was dissolved in 100 μL of Dimethyl Sulfoxide (DMSO). After the solution was gently shaken for 10 minutes using an Eppendorf Mix Mate (Eppendorf, GRE), the absorbance was detected at 490 nm (D490) on a BioTek ELx800 (BioTek, Vermont, USA).

### Colorectal Cell Transwell Migration and Invasion Assay

The chemotactic motility of colorectal cells was determined using transwell migration chambers with 6.5-mm-diameter polycarbonate filters. In brief, the cells were pretreated with IL-6 or AG490 for 48 hours as indicated. As the invasion assay, the bottom chambers were filled with 30μL Matrigel allowed to set. Thereafter, the bottom chambers were filled with 500 μL complete medium. Colorectal cancer cells (5 × 10^4^ per well) were seeded in the top chambers in 200 μL of serum-free medium. The cells were allowed to migrate for 24 hours or invade for 48 hours. Nonmigrated or noninvaded cells were removed with cotton swabs, and migrated or invaded cells were fixed with ice-cold ethanol and stained with 0.01% crystal violet. Images were captured at a ×100 magnification.

### Western Blot Analysis

Whole cells were lysed with RIPA buffer. Protein lysates were separated on a 10% Dodecyl Sulfate,Sodium Salt (SDS)-Polyacrylamide Gel Electrophoresis (SDS-PAGE) gel and electroblotted onto a Polyvinylidene Fluoride (PVDF) membrane (Millipore, USA). After the membrane was incubated with primary antibodies overnight, the secondary antibodies were added and incubated at room temperature for 2 hours. After washing with 0.1%Tween-20 in Tris-buffered saline (TBST) 3 times, an enhanced chemiluminescence detection kit Hongye, CHINA was used. β-actin was used as a loading control, and the bands were assessed with a C-DiGit Blot Scanner (LI-COR Biosciences UK Ltd, Cambridge, UK) and analyzed using ImageJ (v1.48u).

### Three-Dimensional Cultures

Coverslips were coated with 30 μL of Matrigel in 96-well plates. After 4 hours at 37°C, the Matrigel had set, and CRC cells in complete medium were seeded onto the gel and cultured at 37°C for 24 hours. Vascular networks were then filmed under a phase-contrast microscope.

### Immunofluorescence Double Staining

Colorectal cancer cells were cultured on sterile glass cover slips 1 day prior to staining. The cells were fixed with cold methanol for 10 minutes, washed with Phosphate-Buffered Saline (PBS) for 5 minutes and with 0.2% Triton for 30 minutes, and then blocked with 5% Bovine Serum Albumin (BSA) for 1 hour. The slips were incubated overnight with primary antibodies at 4°C. The slides were washed in PBS and labeled with specific secondary antibodies for 1 hour in the dark. The cells were counterstained with 4′,6-diamidino-2-phenylindole (DAPI) and then viewed with a fluorescence microscope.

### Ethics Statements and Consent to Participate

The study protocol was approved by the ethics committee of Tianjin Medical University (TMUhMEC2015006), and informed consent was obtained from each patient and their relatives.

### Statistical Analysis

The data are expressed as the means (standard deviation). A 1-way analysis of variance was used for statistical analyses. SPSS software version 21.0 (SPSS Inc, Chicago, Illinois) was used. A value of *P* < .05 was considered significant.

## Results

### Expression of p-STAT3 in CRC Tissues Is Associated With Clinicopathological Parameters and Prognosis

**Figure 1.**
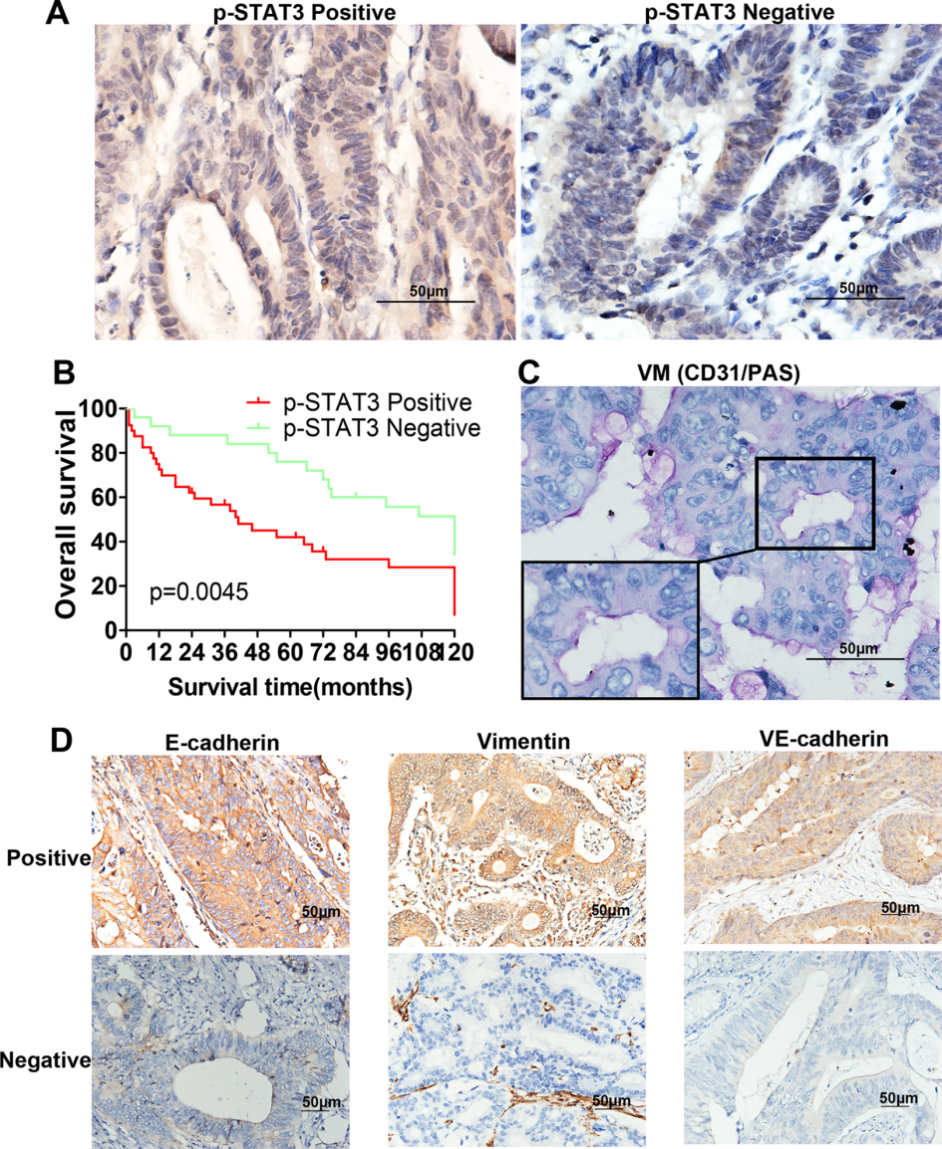
The expression of the proteins on colorectal cancer (CRC) specimens and Kaplan-Meier analysis. A, Positive p-STAT3 expression in CRC specimens and negative p-STAT3 expression for comparison (magnification, ×400). B, CD31/PAS double staining displayed vasculogenic mimicry (VM) channels in CRC specimens (magnification, ×400). C, Kaplan-Meier analysis showed that the patients with p-STAT3-positive samples displayed poorer prognosis (*P* = .0045). D, Positive E-cadherin, vimentin, VE-cadherin expression in CRC specimens, and negative E-cadherin, vimentin, VE-cadherin expression for comparison (magnification, ×200).

As shown in Figure 1A, p-STAT3 was predominantly localized in the nucleus of CRC cells, partially expressed in cytoplasm, and the negative staining of p-STAT3 protein is shown for comparison. Under high-power magnification, 10 random fields from each specimen were selected, and >500 cells were assessed to determine the percentage of positive cells. Percentages ≥10% were considered positive samples. Immunohistochemistry (IHC) analysis of 65 cases showed that 40 samples had strong p-STAT3 expression and the other 25 samples had weak p-STAT3 expression. As shown in Table 1, p-STAT3 expression was significantly higher in advanced-stage carcinomas (TNM stages III and IV, 29/40) than in early-stage carcinomas (TNM stages I and II, 11/25; *P* = .015). Moreover, the p-STAT3-high and p-STAT3-low samples showed significant differences in metastasis (*P* = .040). Finally, a Kaplan-Meier survival analysis indicated that the p-STAT3-high group had poor overall survival compared with the p-STAT3-low group (*P* = .0045; Figure 1B). Therefore, we concluded that the expression of p-STAT3 was significantly correlated with tumor metastasis, TNM stage, and poor prognosis but not with age, gender, tumor size, or differentiation grade.

**Table 1.** Correlation Between p-STAT3 Expression and Clinicopathologic Characteristics of Patients With CRC.

Factors	Summation	p-STAT3 Expression		χ^2^	*P*
		Positive (%)	Negative (%)		
Age, years				0.78	.385
≥45	60	36	24		
<45	5	4	1		
Gender				0.157	.692
Male	28	18	10		
Female	37	22	15		
Tumor size (diamater, cm)				0.745	.45
≥5	33	22	11		
<5	32	18	14		
Tumor stage				6.796	.015^a^
I/II	25	11	14		
III/IV	40	29	11		
Differentiation grade				1.414	.363
I	18	9	9		
II	27	18	9		
III	20	13	7		
Metastasis				4.745	.040^aa^
Negative	28	13	15		
Positive	37	27	10		

Abbreviations: CRC, colorectal cancer.

^a^Significantly different.

### Expression of p-STAT3 Is Correlated With the Presence of VM and the Expression of the VM-Associated Protein VE-Cadherin, the EMT-Associated Protein E-Cadherin, and Vimentin in CRC Tissues

CD31/PAS double staining was used to identify VM in tumors, a method that has been used in many studies.^3,18,19^ As shown in Table 2, among the 65 CRC samples, 10 exhibited the formation of vascular-like networks that were CD31 negative and PAS positive and contained red blood cells (Figure 1C). The results showed that 22.5% (9/40) of the samples in the p-STAT3-high group displayed VM, while in the p-STAT3-low group, only 4% (1/25) of the samples showed VM (*P* = .044). Moreover, we found that 65% (26/40) of the p-STAT3-high group overexpressed VE-cadherin compared with 36% (9/25) of the p-STAT3-low group (*P* = .022; Figure 1D). In addition, 48.5% (16/33) of the samples in the p-STAT3-high group were E-cadherin positive compared to the p-STAT3-low group (*P* = .028; Figure 1D). Moreover, a similar phenomenon was observed for vimentin expression (*P* = .026; Figure 1D). Based on these data, we concluded that the expression of p-STAT3 is correlated with the presence of VM and the expression of VE-cadherin, E-cadherin, and vimentin in CRC tissues.

**Table 2.** Correlation Between p-STAT3 Expression and VM Formation, VE-Cadherin, E-Cadherin, and Vimentin Expression in CRC.

Factors	Summation	p-STAT3		χ^2^	*P*
		Positive	Negative		
VM				4.045	.044^a^
Positive	10	9	1		
Negative	55	31	24		
VE-cadherin				5.206	.022^a^
Positive	35	26	9		
Negative	30	14	16		
E-cadherin				4.826	.028^a^
Positive	33	16	17		
Negative	32	24	8		
Vimentin				4.940	.026^a^
Positive	21	17	4		
Negative	44	23	21		

Abbreviation: VM, vasculogenic mimicry.

^a^Significantly different.

### Determination of the Optimal Concentrations of IL-6 and AG490 for Treating the HCT116 and HT29 CRC Cell Lines by Western Blot Analyses and Cellular Morphological Changes

**Figure 2.**
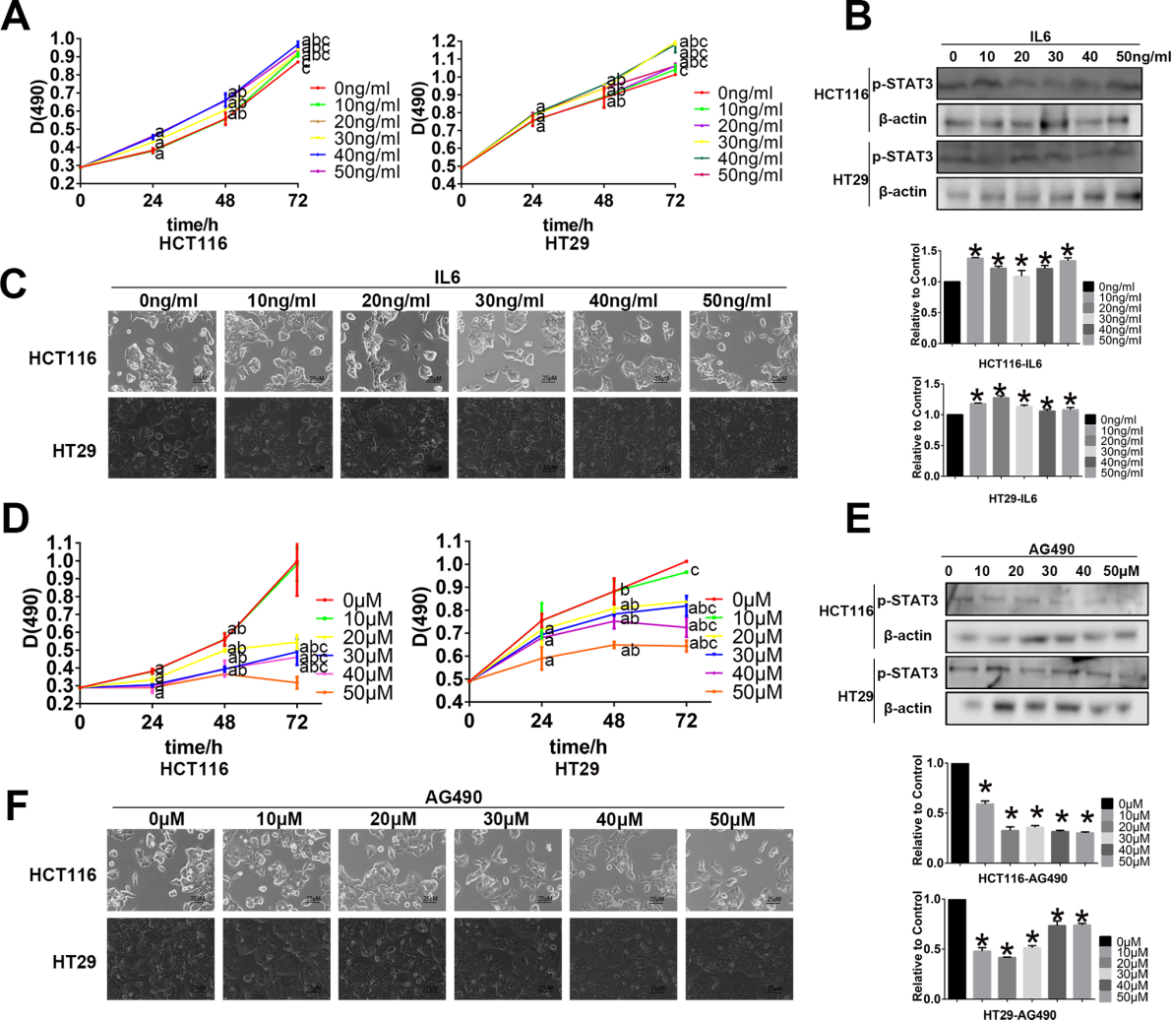
Western blot assay, MTT assay, and cellular morphology to find best diluted concentration of interleukin 6 (IL-6) and AG490. A, The MTT assay shows the cell proliferation with the different diluted concentration of IL-6 treated on HCT116 and HT29 for 24, 48, and 72 hours (^a^ *P* < .05, compared with the control group at the same time; ^b^compared with the same concentration at 24 hours; ^c^compared with the same concentration at 48 hours). B, The Western blot assay shows the change in p-STAT3 activation with the different diluted concentrations of IL-6 treated on HCT116 and HT29 for 48 hours. C, The cellular morphology of different dilution of IL-6 treated on HCT116 and HT29 for 48 hours (magnification, ×400). D, The MTT assay show the cell proliferation with the different diluted concentration of AG490 treated on HCT116 and HT29 for 24, 48, and 72 hours.(^a^ *P* < .05, compared with the control group at the same time; ^b^compared with the same concentration at 24 hours; ^c^compared with the same concentration at 48 hours). E, The Western blot assay show the change in p-STAT3 activation with the different diluted concentration of AG490 treated on HCT116 and HT29 for 48 hours. F, The cellular morphology of different dilutions of AG490 treated on HCT116 and HT29 for 48 hours (magnification, ×400).

We examined the effects of different concentrations of IL-6 and AG490 on HCT116 and HT29 cell lines for 48 hours. We found that when HCT116 cells were treated with IL-6, p-STAT3 was significantly upregulated, and the concentration of 10 ng/mL had the largest effect in the HCT116 cell line (Figure 2B). Furthermore, as MTT assay showed, 10 and 20 ng/mL IL-6 for 24 or 48 hours, there is no significant difference between the control group and experimental group, which can eliminate the effect of cell proliferation (Figure 2A). Morphologically, these cells become more thin and long when treated with 20 ng/mL IL-6 than with 10 ng/mL (Figure 2C). So we chose 20 ng/mL as the IL-6 treatment concentration for the HCT116 cell line. For the HT29 cell line, we chose the same 20 ng/mL as IL-6 concentration which resulted in high-p-STAT3 expression, and MTT assay showed, 10 and 20 ng/mL IL-6 for 24 or 48 hours, there is no significant difference between the control group and experimental group, which can eliminate the effect of cell proliferation (Figure 2A, B). Using the same method, MTT assay showed 10 μM AG490 would not significantly affect the 2 cell proliferation (Figure 2D), and Western blot gave a relatively significant inhibition of p-STAT3 activation (Figure 2E), so we chose 10 μM as the optimal AG490 concentration for the HCT116 and HT29 cell lines, and we can get a slight change in the cell morphology for the cells that became a little shorter (Figure 2F).

### p-STAT3 Activation Resulted in EMT in CRC Cells

**Figure 3.**
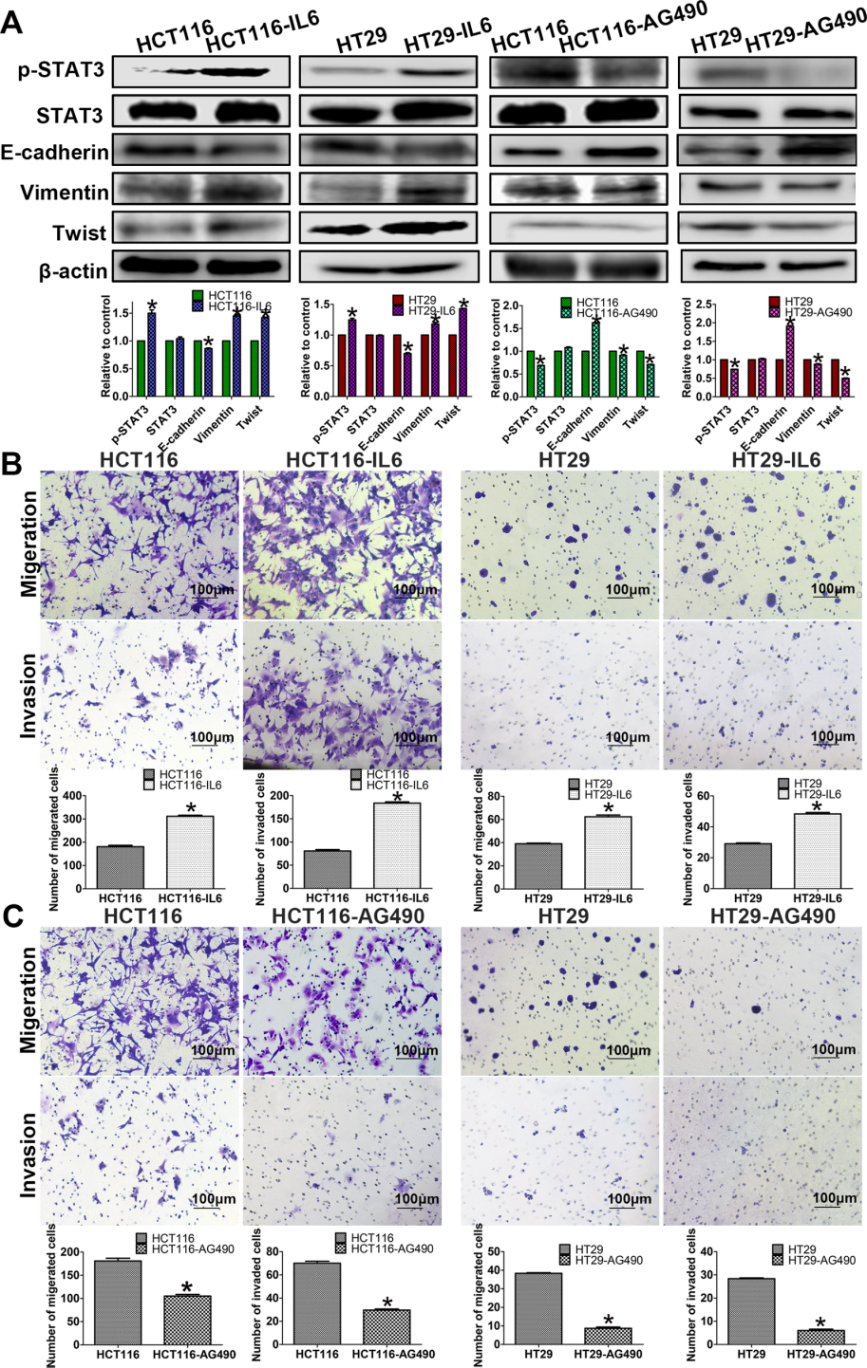
The related protein expressions and cell migration and invasion. A, Western blot assays show that IL-6 induces p-STAT3 activation on the 2 cell lines, E-cadherin downexpressed, and vimentin, Twist upexpressed. And AG490 inhibit p-STAT3 activation on the 2 cell lines, E-cadherin upexpressed, and vimentin, Twist downexpressed. B and C, Transwell assays were performed in the cell lines to indicate that p-STAT3 activation could affect migration and invasion (magnification, ×100). B, The migration and invasion abilities of HCT116 and HT29 cells were increased following the induction of IL-6. C, The migration and invasion abilities of HCT116 and HT29 cells were decreased following AG490 treatment.

Western blot analyses showed that the IL-6-induced HCT116 cells had significantly decreased the expression of the epithelial marker E-cadherin and increased the expression of the mesenchymal marker vimentin as well as transcription factor Twist compared with the control cells (Figure 3A). Similarly, AG490-treated HCT116 cells and AG490-treated HT29 cells showed epithelioid changes, including decreased vimentin and Twist expression with increased E-cadherin expression, as determined by Western blot analyses (Figure 3A).

### p-STAT3 Activation Enhances the Migration and Invasion Abilities of CRC Cells *In Vitro*

Both EMT and VM formation are associated with cell migration and invasion; therefore, transwell assays were performed to investigate the effects of p-STAT3 activation in CRC cells. The results suggested that compared with control cells, IL-6-treated HCT116 and HT29 cells had increased migration and invasion abilities (Figure 3B). Additionally, significant differences were found between control and AG490-treated HCT116 cells and between control and AG490-treated HT29 cells (Figure 3C).

### p-STAT3 Activation Regulates the VM Formation of CRC Cells *In Vitro* and Upregulates the Expression of VE-Cadherin

**Figure 4.**
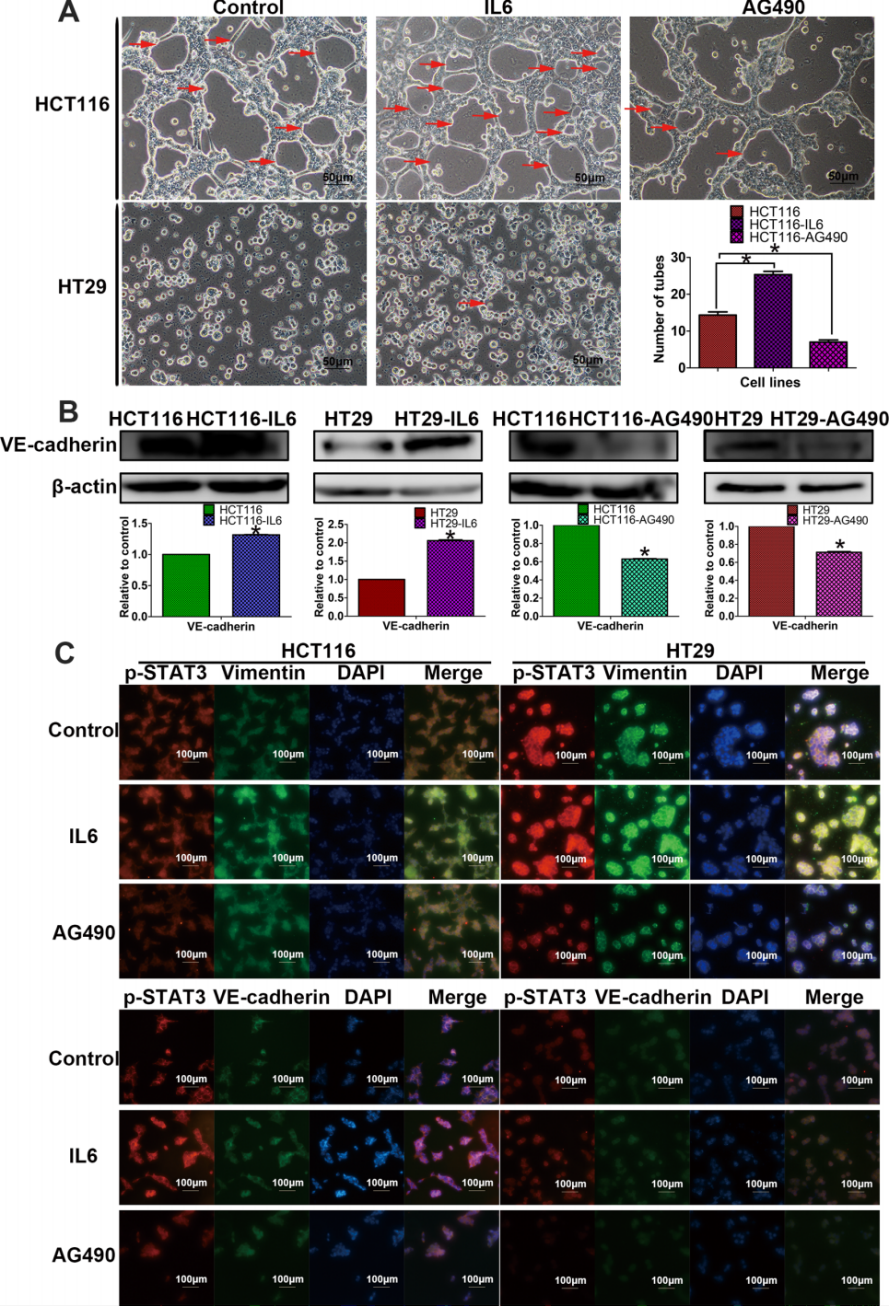
p-STAT3 activation promotes vasculogenic mimicry (VM) by inducing vimentin. A, 3-D culture assays suggested that p-STAT3 activation has positive influence on the VM (magnification, ×200). Interleukin 6 (IL6)-induced HCT116 form more vascular structures than control, and HT29 cells have a trend to form a tube-like structure. AG490-inhibited HCT116 form less vascular structures than control. B, Western blot assays show that IL-6 induces p-STAT3 activation and VM marker VE-cadherin upexpressed; AG490 inhibits p-STAT3 activation and VE-cadherin downexpressed. C, Immunofluorescence double staining assays indicated that p-STAT3 activation may induce VM formation by inducing epithelial-to-mesenchymal transition (EMT) marker vimentin, not directly affecting on VM marker VE-cadherin (magnification, ×100).

We initially used a well-established *in vitro* 3-D culture model to investigate CRC VM formation. We used HCT116, which can form typical vessel-like tubes, to investigate the effect of AG490 on VM formation. In addition, we used the 2 cell lines to observe the effect of IL-6 on VM formation. We observed that IL-6-induced HCT116 cells formed more tubular structures, while AG490-treated HCT116 cells formed fewer tubular structures than control. HT29 cells formed few tubular structures, and we also observed that using IL6-induced HT29 cells has a trend to form a tube-like structure. The above results indicated that p-STAT3 is a requirement for VM formation in HCT116 and HT29 cells (Figure 4A). Additionally, compared with the control cells, the IL-6-induced HCT116 cells and IL-6-induced HT29 cells showed higher VE-cadherin expression. Conversely, AG490-treated HCT116 and HT29 cells expressed less VE-cadherin than the control cells (Figure 4B).

### p-STAT3 Activation Indirectly Induces VM Formation by Upregulating Vimentin Expression, Activating the EMT Process

Immunofluorescence double staining assays were used to explore whether p-STAT3 activation directly induces VM formation by upregulating the VM marker VE-cadherin or indirectly affects VM by first inducing EMT. The results suggested that IL6-induced p-STAT3 activation is associated with the upregulation of the EMT marker vimentin in both HCT116 and HT29 cells (Figure 4C). AG490 treatment indicated a similar link between p-STAT3 and vimentin. However, when analyzing VE-cadherin expression, the results showed that when p-STAT3 is activated, the expression of VE-cadherin does change, but there is no significant direct correlation between p-STAT3 and VE-cadherin coexpression in the cells (Figure 4C). As a consequence, we conclude that p-STAT3 activation may indirectly induce VM formation by first upregulating vimentin expression and activating the EMT process.

## Discussion

It has been demonstrated that p-STAT3 activation is required to sustain EMT-associated phenotypes in ovarian and breast cancers.^20,21^ Moreover, p-STAT3 activation is associated with lung cancer.^22^ Our results support the potential role of p-STAT3 activation in promoting metastasis and EMT and inducing the formation of VM patterns in CRC. From our human CRC samples, we found that p-STAT3 expression is positively correlated with metastasis. We also found that in CRC cell lines, activated p-STAT3 can promote cell invasiveness and metastasis. These results are consistent with other researchers’ findings, which associate p-STAT3 activation with CRC progression and metastasis.^23,24^ Our individual patient data and 3-D culture assays demonstrate a link between p-STAT3 expression and VM formation.

Vasculogenic mimicry has been reported in many malignant tumors and is associated with high tumor grade and more aggressive, poorly differentiated and highly metastatic tumors.^5–9^ Our previous studies have shown that VM was related to poor prognosis, progression, and metastasis in CRC.^25,26^ Vasculogenic mimicry -forming tumor cells form vessel-like structures by altering their cell phenotype and markers into a pattern similar to vascular endothelial cells.^27^ Previous evidence has shown that the activation of EMT may be associated with VM formation.^25,27^

Epithelial-to-mesenchymal transition is a process by which epithelial cells acquire the characteristics of mesenchymal cells.^28^ Our group has proposed that EMT is an important pathway to VM formation.^19,29^ One study reported that STAT3 binds to the promoter of Hypoxia-Inducible Factor 1α (HIF-1α), indicating that p-STAT3 activation could regulate hypoxia-induced EMT.^30^ Another group of researchers came to a similar conclusion as ours that IL-6 induces STAT3 phosphorylation and further causes EMT changes in cancer cells.^31^

Interleukin 6 is considered to be an inducer of p-STAT3.^32^ In our present study, we use IL-6 to induce p-STAT3 activation. Our results show that activated p-STAT3 can lead to reduced expression of E-cadherin and increased expression of vimentin, the 2 vital proteins of EMT. In addition, we observed increased migration and invasion abilities in CRC cells treated with IL-6. These findings are in accordance with previous studies demonstrating that IL-6 treatment can activate p-STAT3, decrease E-cadherin expression, and increase vimentin expression in other cancer cell lines, such as lung cancer cell lines^33^ and breast cancer cell lines.^34^

Jak is responsible for the tyrosine phosphorylation of STAT3 in response to extracellular signals and oncogenes.^35^ AG490, a micromolecular Jak inhibitor, can block the constitutive activation of p-STAT3.^36^ In our Western blot assays, we observed that when p-STAT3 expression was inhibited by AG490, E-cadherin expression was upregulated, and vimentin expression was downregulated. In addition, transwell assays indicated that AG490-treated cells demonstrated less migration and invasion than control cells. These results also indicate that p-STAT3 activation is positively correlated with EMT and colorectal carcinoma metastatic capability as well as invasion ability.

VE-cadherin has been used as a critical biomarker of VM formation.^37^ When we treated the HCT116 and HT29 CRC cell lines with IL-6 or AG490, the Western blot assay results showed that VE-cadherin expression changed with the expression of p-STAT3 at the protein level. Some researchers have used IL-6 and AG490 to treat other cancer cell lines, and their results were similar to ours, in that p-STAT3 activation affected the expression of VE-cadherin and subsequently regulated migration and invasion as well as EMT.^33,38^ Moreover, 3-D culture assays showed that p-STAT3 activation induces the formation of vessel-like structures.

Based on the above results, we wanted to explore the potential connection between p-STAT3 activation and VM formation and determine whether p-STAT3 activation directly affects the VM-related protein VE-cadherin or indirectly regulates and controls VM by affecting EMT-related proteins. We chose the vital marker vimentin as the target of EMT. According to the results of our immunofluorescence double staining experiments in cancer cell lines treated with IL-6 or AG490, we found that p-STAT3 activation is more closely linked with vimentin expression than VE-cadherin expression. And we can make a preliminary conclusion that p-STAT3 activation can induce EMT by raising vimentin expression, and with the process of EMT, VM may be easier to form. According to the Western blot assay, although p-STAT3 activation can induce VE-cadherin protein upregulation, p-STAT3 cannot induce VE-cadherin upregulation directly due to the immunofluorescence double staining assay. We speculated that there may exist some other pathways between p-STAT3 activation and VE-cadherin, and the means of p-STAT3 activation may accrete the formation of VM is not from directly inducing the marker VE-cadherin expression, but it is caused by the process of EMT.

In conclusion, the clinicopathological evidence showed a correlation between p-STAT3 activation and malignant CRC progression. These results suggested that p-STAT3 may be a diagnostic marker of poor prognosis. We demonstrated that p-STAT3 activation plays a vital role in promoting VM formation in HCT116 and HT29 cells. According to our experiments, we found that p-STAT3 activation can induce EMT by upregulating snail and vimentin and downregulating E-cadherin. Above all, our experimental results suggested that p-STAT3 activation may indirectly induce VM. It may firstly upregulate vimentin expression, activating the EMT process, and subsequently regulating VM formation. Therefore, preventing p-STAT3 activation might serve as a therapeutic method of reducing EMT and VM formation, thereby improving the prognosis of CRC.

## Supplementary Material

Supplementary material

## Abbreviations

CRC: colorectal cancer
EMT: epithelial-to-mesenchymal transition
IL-6: interleukin 6
VM: vasculogenic mimicry.
