# Supplementary material for: Phosphorylation of STAT3 Promotes Vasculogenic Mimicry by Inducing Epithelial-to-Mesenchymal Transition in Colorectal Cancer
Source: Technol Cancer Res Treat. 2017 Nov 22;16(6):1209–19. doi: 10.1177/1533034617742312 (PMC5762092; doi:10.1177/1533034617742312)
Supplement: Supplementary material [file Supplemental_Material.pdf]

Phosphorylation of STAT3 promotes vasculogenic mimicry by inducing epithelial-to-mesenchymal transition in colorectal cancer

---

Supplementary Materials

The detail of the primary antibodies for immunohistochemical Staining and Western blot analysis.

Vimentin antibody(Cat. #ab92547),Twist antibody(Cat. #ab50581)and VE-cadherin antibody(Cat. #ab33168) were purchased from abcam co.(Cambridge, UK). STAT3(Cat. #sc-8019),β-actin antibody(Cat. #sc-47778) were purchased from santa cruz biotechnology(CA, USA).

P-stat3(Cat. #9145s) were purchased from Cell Signaling Technology (USA).

The detail of the primary antibodies for Immunofluorescence double staining.

Vimentin antibody(Cat. #sc-6260)and VE-cadherin antibody(Cat. #sc-52751) were purchased from santa cruz biotechnology(CA, USA).

The secondary antibodies were purchased from Zhongshan Golden Bridge Biotechnology Co., Ltd. (Beijing, China).

Matrigel was purchased from BD Biosciences (NY, USA).

Recombinant Human Interleukin-6 (IL6) (Cat. #200-06)was purchased from PeproTech(USA).

AG490(Cat. #S1143) was purchased from Selleck(USA).

MTT Kit was purchased from TIANGEN (China).
